# Evaluation of Stainless Steel Miniplates in Mandibular Fractures Using Scanning Electron Microscopy and Energy-Dispersive X-ray Analysis: A Retrospective Observational Study

**DOI:** 10.7759/cureus.91650

**Published:** 2025-09-05

**Authors:** Priya Manimala, Thomson Dcruz, Shubanshi Kangloo, Mangal More, Saurabh Gohil, Khushal Desai

**Affiliations:** 1 Oral and Maxillofacial Surgery, YMT Dental College and Hospital, Navi Mumbai, IND; 2 Oral and Maxillofacial Surgery, Datta Meghe Institute of Medical Sciences, Wardha, IND

**Keywords:** energy dispersive x-ray analysis(edx), mandibular fractures, scanning electron microscope(sem), stainless steel miniplates, tribocorrosion

## Abstract

Stainless steel miniplates are widely used for mandibular fracture fixation due to their affordability and stability, yet concerns remain regarding long-term biocompatibility and corrosion-related degradation. This retrospective observational study compared the surface characteristics and elemental composition of stainless steel miniplates retrieved after 6-12 months (Group A; mean 8.4 months) and 24 months (Group B; mean 24.2 months) using Scanning Electron Microscopy (SEM) and Energy Dispersive X-ray (EDX) analysis, and correlated these findings with clinical outcomes. Thirty patients were divided equally between the two groups. Clinical parameters, including pigmentation, erythema, suppuration, granulation tissue, tenderness, and plate exposure, were recorded and compared between groups. Statistical analysis was performed using the Chi-square test with phi (φ) effect sizes. Group B exhibited significantly higher corrosion (p = 0.020, φ = 0.42), surface roughness (p = 0.020, φ = 0.42), pigmentation (p = 0.002, φ = 0.60), erythema (p = 0.048, φ = 0.36), suppuration (p = 0.001, φ = 0.71), and granulation tissue (p = 0.032, φ = 0.30) compared with Group A, while no significant difference was observed in plate exposure (p = 0.25). EDX analysis revealed greater release of nickel, chromium, and iron in Group B, although binary distribution analysis of elements was not statistically significant (p = 0.157). These findings suggest that prolonged retention of stainless steel miniplates beyond one year increases tribocorrosion and metal ion release, correlating with higher complication rates. Early removal within 6-12 months, when clinically feasible, may help minimize corrosion-related risks and improve patient outcomes.

## Introduction

Tribology, the study of friction, wear, and lubrication, and corrosion, the chemical degradation of materials, together form the field of “tribocorrosion.” This interdisciplinary term describes the degradation of material surfaces due to simultaneous mechanical loading and environmental (chemical) corrosion. Tribocorrosion can be categorized into two areas of study: industrial systems and living biological systems [1,2].

Since 1978, when Champy updated the techniques proposed by Michelet FX et al., internal fixation devices have gained wider acceptance in the management of maxillofacial trauma, orthognathic surgery, and reconstructive procedures [3]. Miniplate osteosynthesis is now widely accepted as a reliable method for providing stable bone fixation [4]. Stainless steel has been one of the primary materials used in maxillofacial surgery; however, concerns have emerged in recent years regarding metal ion release from these implants [4]. Despite its good corrosion resistance, stainless steel may still release low levels of metal ions when in contact with biological fluids [5].

The Strasbourg Osteosynthesis Research Group (SORG) has recommended the removal of non-functional plates when doing so does not pose a significant surgical risk [6]. Wear and corrosion of implant materials can lead to the formation of micro- and nano-sized metal particles, triggering adverse tissue reactions and potentially leading to implant failure [7]. These particles may originate during the insertion and removal of plates or from interactions at the plate-screw interface [8].

Given these concerns, the primary objective of this study was to evaluate and compare the surface morphology and elemental composition of stainless steel miniplates retrieved at two different postoperative intervals using Scanning Electron Microscopy (SEM) and Energy Dispersive X-ray (EDX) analysis. The secondary objective was to determine whether these surface and compositional changes correlated with clinical complications observed in the patients. This dual approach allows both a material-level assessment of tribocorrosion and a clinically relevant understanding of its potential implications for patient care.

## Materials and methods

This retrospective observational multicenter study was conducted at a recognized dental college between January 2018 and December 2020, following approval from the Institutional Ethical Committee (approval number: RCDS/MPMSU/MDS/2018-19/24409, approved on March 25, 2019). Written informed consent was obtained from all patients prior to treatment.

Study groups

Group A

Miniplates retrieved after 6-12 months (mean 8.4 months)

Group B

Miniplates retrieved after 24 months (mean 24.2 months)

The sample size was determined by the total number of eligible cases treated during the study period; no formal a priori power calculation was performed due to the retrospective nature of the study. SEM/EDX analyses were conducted without blinding of the analysts to the retrieval interval. All retrieved plates underwent a standardized cleaning protocol (gentle brushing with saline) before SEM imaging to ensure surface visibility. While this may have removed some superficial corrosion, the process was consistent across all samples and is acknowledged as a limitation.

The inclusion and exclusion criteria for participant selection are summarized in Table 1.

**Table 1:** Inclusion and exclusion criteria.

Criteria Type	Details
Inclusion	Patients aged 18-50 years who underwent open reduction and internal fixation using stainless steel miniplates, with subsequent plate removal due to infection, plate exposure, or patient request.
Exclusion	Patients with underlying systemic conditions affecting wound healing, or those who had undergone prior surgeries using other types of implants.

A total of 30 patients (n = 15 per group) were included. The mean age was 32.6 years (range: 18-50), with a male-to-female ratio of 2:1, reflecting the higher prevalence of mandibular fractures in males. The distribution of fracture sites included the symphysis/parasymphysis (40%), body (33%), and angle (27%). Indications for plate removal were infection (43.3%), pain (33.3%), plate exposure (13.3%), and patient request (10%). Both groups were comparable at baseline in terms of age, gender distribution, and fracture site, ensuring that differences observed were primarily attributable to the duration of plate retention.

Surgical procedure

All surgical procedures were performed by a single experienced oral and maxillofacial surgeon, who was calibrated for the procedure to minimize inter-operator variability. Surgical intervention was determined by the type and severity of infection and the timing of plate retrieval. Procedures were performed under local or general anesthesia, maintaining strict aseptic protocols, including intraoral flushing with 5% povidone-iodine and normal saline.

For symphysis, parasymphysis, and body fractures, a vestibular incision was used, while Ward’s incision was performed for angle fractures. After flap elevation, screws (1.5 mm or 2 mm) were removed with a screwdriver, and plates were retrieved. Closure was achieved using 3-0 vicryl or silk sutures, and an adhesive pressure bandage was applied. Retrieved plates were cleaned with saline, dried, and stored in amber-colored formalin bottles before laboratory analysis.

SEM and EDX analysis

The surface morphology of retrieved plates was examined using a Field Emission Scanning Electron Microscope (ULTRA Plus; Carl Zeiss AG, Oberkochen, Germany). The microscope is equipped with multiple detectors, including in-lens (surface structure), SE2 (topography), EsB (pure material contrast), AsB (channeling contrast), and CL (luminescent materials), along with Scanning Transmission Electron Microscopy (STEM) mode. It provides a resolution up to 0.8 nm with a magnification range of 12-100,000× (SE) and 100-100,000× (BSE).

Elemental composition was analyzed using EDX spectroscopy (Oxford Instruments, Abingdon, UK), which identifies localized elemental release (nickel, chromium, iron, etc.) from the implant surface into adjacent tissues.

Statistical analysis

Clinical parameters, including pigmentation, erythema, suppuration, granulation tissue, plate exposure, and tenderness, were analyzed using the Chi-square (χ²) test of independence to compare categorical differences between Groups A and B. This test was chosen because the outcomes were binary (presence/absence) and compared between two independent groups. Effect size was measured using the phi coefficient (φ), interpreted as small (≈0.10), medium (≈0.30), or large (≥0.50). A p-value < 0.05 was considered statistically significant. All statistical analyses were performed using IBM SPSS Statistics for Windows, Version 25.0 (IBM Corp., Armonk, NY, USA).

## Results

Clinical, macroscopic, SEM, and EDX analyses revealed significant differences between the two groups in terms of surface roughness, corrosion, and elemental composition. These macroscopic parameters are summarized in Table 2.

**Table 2:** Macroscopic findings.

Variable	Group A (n = 15)	Group B (n = 15)	χ² (df = 1)	p-value	Effect Size (φ)
Pigmentation	0	8	10.9	0.002	0.6
Erythema	7	12	3.98	0.048	0.36
Suppuration	2	9	15	0.001	0.71
Plate exposure	4	7	1.29	0.25	0.21
Granulation tissue	11	15	4.61	0.032	0.3
Tenderness	10	10	0	1	0

**Figure 1:**
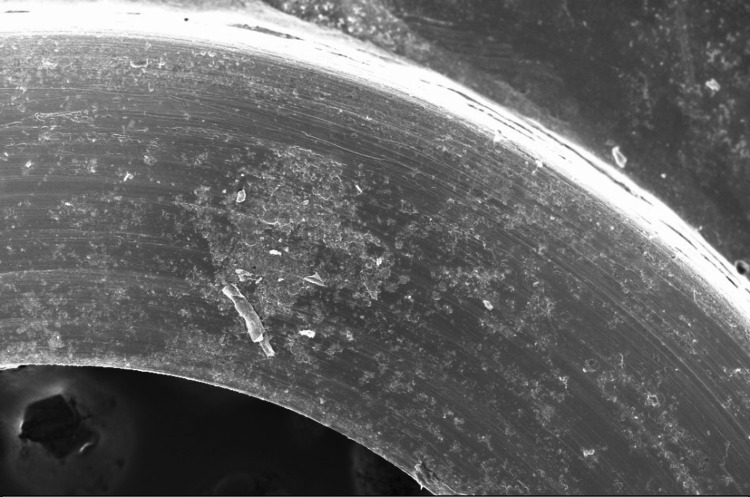
Surface appearance of a stainless steel miniplate retrieved 8 months after surgery. Shallow surface irregularities in the form of pits and craters extend across the left corner. The arrow indicates a small crack. Field width: 100 µm (low vacuum SEM, ×250).

The SEM results showed that Group A (plates removed after 6-12 months) exhibited minimal surface roughness and localized minor pitting at the plate-screw interface, as illustrated in Figure 1.

**Figure 2:**
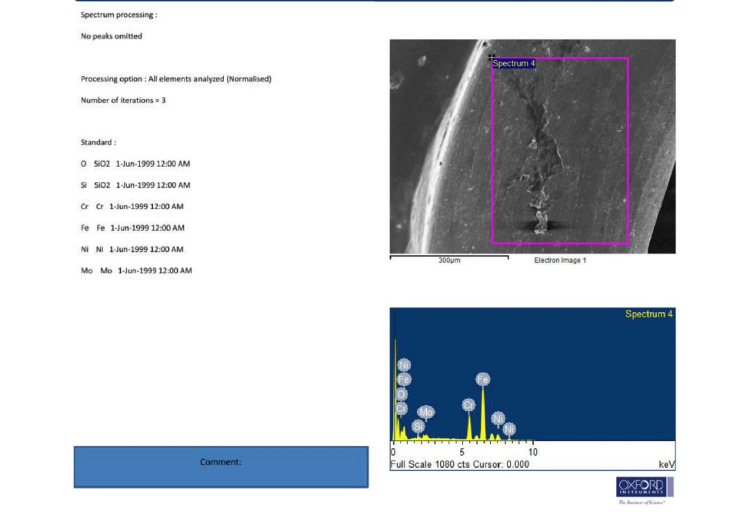
All values obtained through EDX of plates retrieved after 8 months (Spectrum 4) regarding the composition of miniplates were identical and comparable to control stainless steel miniplates. The atomic percentage of iron indicates leaching of Fe into the surrounding tissues, causing inflammatory reactions. EDX: Energy Dispersive X-ray.

EDX analysis of the same Group A plates revealed low levels of metal ion release (nickel, chromium, and iron), indicating minimal degradation. The EDX data are presented in Figure 2.

**Figure 3:**
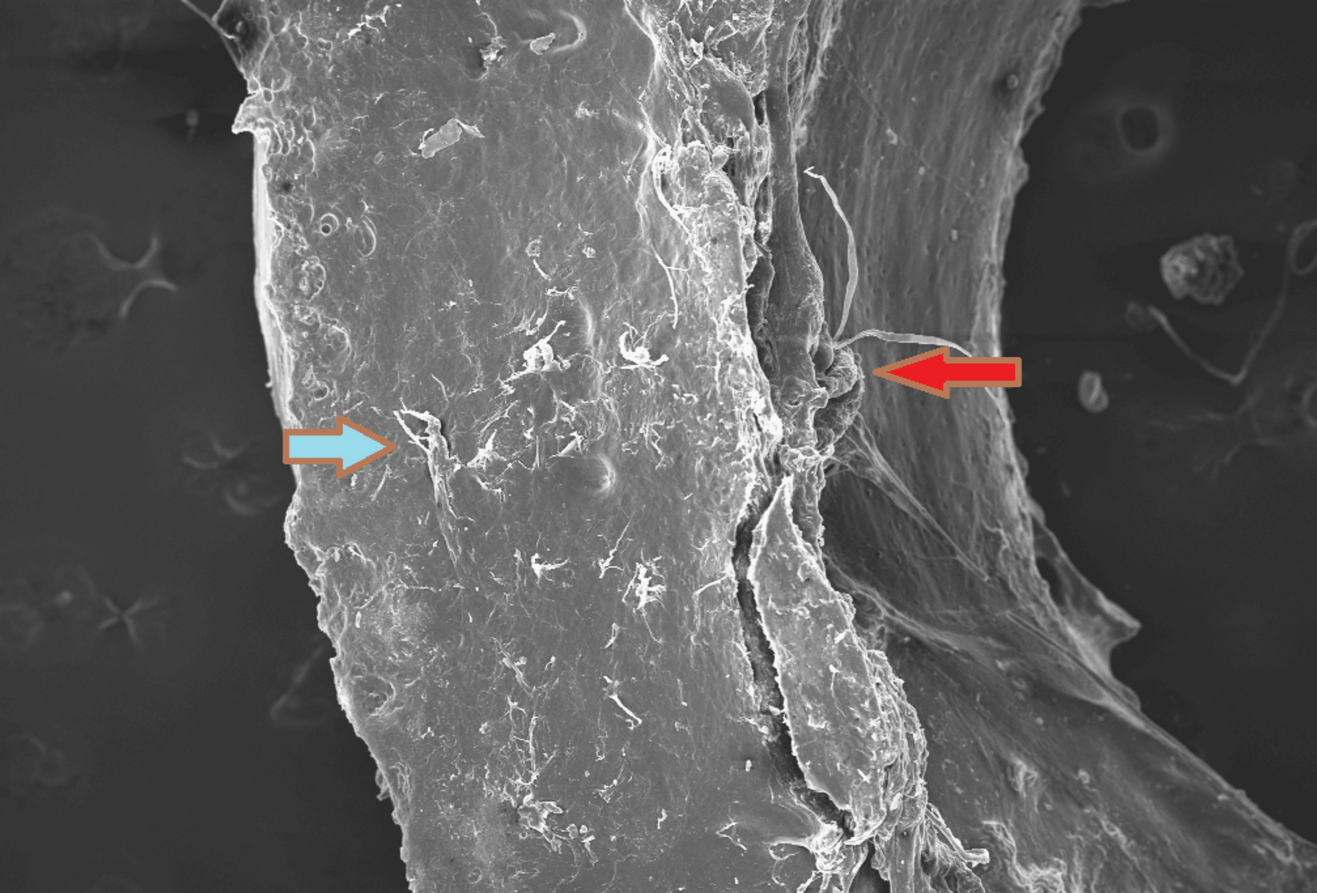
SEM view of a stainless steel plate. The red arrow indicates groups of randomly arranged polymorphous defects and the presence of pits and craters across the surface of the plate. The blue arrow indicates surface cracks. Field width: 9.1 mm (low vacuum SEM, ×100). SEM: Scanning Electron Microscopy.

In contrast, Group B (plates removed after 2 years) exhibited significant corrosion, with widespread surface degradation, particularly at the screw-plate interface, as demonstrated in Figure 3.

**Figure 4:**
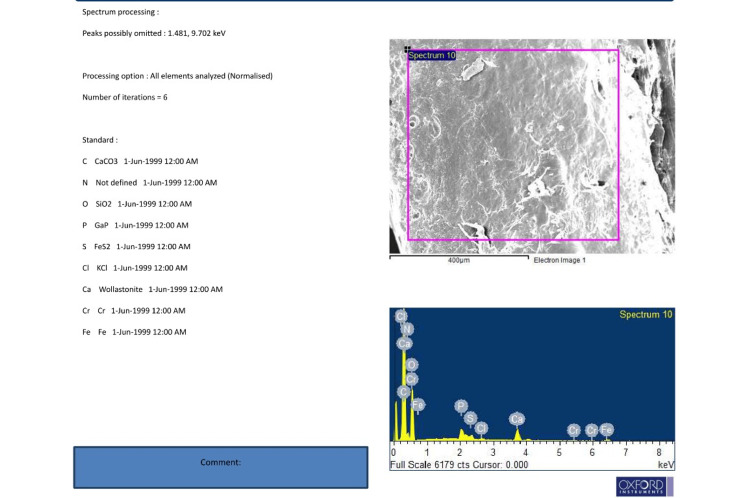
All values obtained through EDX under Spectrum 10 in plates retrieved after 14 years show the presence of chloride, confirming pitting corrosion of the protective layer of stainless steel. It can be concluded that the formation of pits and the presence of stress can lead to Cl-SCC (Stress Corrosion Cracking) failure of the plates. In addition, sulfide ions can significantly accelerate SCC failure. The atomic percentage of iron indicates leaching of Fe into the surrounding tissues, causing inflammatory reactions. EDX: Energy Dispersive X-ray.

EDX analysis of Group B detected substantially higher levels of nickel, chromium, and iron, suggesting considerable material degradation and ion release. These findings are shown in Figure 4.

A comparative summary of SEM-based parameters between the groups is presented in Table 3.

**Table 3:** SEM findings. SEM: Scanning Electron Microscopy.

Variable	Group A (n = 15)	Group B (n = 15)	χ² (df = 1)	p-value	Effect Size (φ)
Corrosion	8	15	5.4	0.02	0.42
Surface roughness	8	15	5.4	0.02	0.42
Color changes	1	6	4.65	0.03	0.39

Effect size interpretation for φ

Small: ≥ 0.10

Medium: ≥ 0.30

Large: ≥ 0.50

To statistically compare the elemental composition between the two groups, a Chi-square test for independence was performed using binary presence/absence values of 12 elements in Spectrum 4 (Group A) and Spectrum 10 (Group B). The results are shown in Table 4.

**Table 4:** EDX findings. EDX: Energy Dispersive X-ray.

Statistic	Value
Chi-square (χ²)	2
Degrees of freedom	1
p-value	0.157
Effect size (φ)	0.29

There was no significant difference in the distribution of elements between the two spectra (p = 0.157). However, elements such as Ni and Mo were detected only in Spectrum 4, indicating localized compositional variation.

## Discussion

Internal fixation of mandibular fractures following the tension band principle was first proposed by Michelet FX et al. and later revised by Champy et al. [9,10]. This technique promotes primary bone healing by providing stable, compressive contact between fracture segments, allowing functional occlusion and mandibular mobility. Stability is a critical factor in preventing infection, particularly in a mobile and load-bearing bone such as the mandible [11].

Stainless steel and titanium remain widely used in maxillofacial surgery because of their mechanical strength and relative biocompatibility. However, they are not biologically inert and can undergo tribocorrosion, leading to the release of metal ions when exposed to the intraoral environment [5]. The literature remains inconclusive regarding the ideal time for miniplate removal. Some studies report removal within 6 months [12], while others suggest longer retention periods of 9-14 months [13,14].

In this study, infection was the most common reason for plate removal (43.3%), followed by pain (33.3%), consistent with previous reports [5]. SEM and EDX analyses confirmed that surface irregularities, corrosion, and metal ion leaching increased with longer retention periods. Plates retrieved at 6-12 months showed only shallow surface pits and cracks, while those retrieved after 24 months demonstrated widespread pitting, polymorphous defects, and evidence of stress corrosion cracking. Similar findings have been documented in earlier retrieval studies [15,16].

EDX analysis revealed higher leaching of iron, chromium, and nickel in plates retrieved after two years, along with the detection of chloride and sulfide ions, which accelerate localized corrosion and stress corrosion cracking. This correlates with reports by Dugal A et al. and Torgersen S et al., who found elevated metal ion release in peri-implant tissues [4,16]. Collectively, these findings indicate that the risk of tribocorrosion-related complications increases with implant duration.

Clinical implications

The findings of this study emphasize that prolonged retention of stainless steel miniplates is associated with increased corrosion, surface degradation, and metal ion release, which may contribute to adverse tissue reactions and implant-related complications. From a clinical standpoint, elective removal of stainless steel miniplates within 6-12 months postoperatively, when fracture healing is complete and removal does not pose significant risk, may help reduce long-term complications. These results also support consideration of titanium as an alternative material due to its superior corrosion resistance and biocompatibility.

Limitations

This study has several limitations. First, the retrospective observational design and small sample size restrict the generalizability of the findings. Second, SEM and EDX analyses were not blinded to group allocation, which could introduce bias. Third, the standardized cleaning protocol before SEM imaging may have altered some superficial corrosion features. Finally, no long-term follow-up of systemic effects from metal ion release was performed. Future prospective studies with larger cohorts, randomization, and extended follow-up are recommended to validate these results and further investigate the biological impact of prolonged implant retention.

## Conclusions

Stainless steel miniplates are effective and commonly used for the internal fixation of mandibular fractures. However, prolonged retention beyond one year increases the risk of tribocorrosion, material degradation, and metal ion leaching, which may lead to adverse tissue responses and implant failure. Based on our findings, it is advisable to remove stainless steel miniplates within 6-12 months postoperatively, provided that their removal does not compromise bone healing or patient safety. Early removal may help minimize corrosion-related complications.

Future studies should explore the performance of alternative materials such as titanium, which exhibits superior biocompatibility and corrosion resistance. Additionally, investigations into the long-term systemic effects of ion release and bioaccumulation from stainless steel implants are warranted.
